# Toxicity effects of di-(2-ethylhexyl) phthalate to *Eisenia fetida* at enzyme, cellular and genetic levels

**DOI:** 10.1371/journal.pone.0173957

**Published:** 2017-03-20

**Authors:** Tingting Ma, Wei Zhou, Li’ke Chen, Longhua Wu, Peter Christie, Haibo Zhang, Yongming Luo

**Affiliations:** 1 Institute of Hanjiang, Hubei University of Arts and Science, Xiangyang, China; 2 Key Laboratory of Soil Environment and Pollution Remediation, Institute of Soil Science, Chinese Academy of Sciences, Nanjing, China; 3 School of Civil Engineering and Architecture, Hubei University of Arts and Science, Xiangyang, China; 4 Shanghai Research Institute of Chemical Industry, Shanghai, China; 5 Key laboratory of Coastal Environmental Processes and Ecological Remediation, Yantai Institute of Coastal Zone Research, Chinese Academy of Sciences, Yantai, China; Jinling Institute of Technology, CHINA

## Abstract

Di-(2-ethylhexyl) phthalate (DEHP) is a dominant phthalic acid ester (PAE) that has aroused public concern due to its resistance to degradation and its toxicity as an endocrine-disrupting compound. Effects of different concentrations of DEHP on *Eisenia fetida* in spiked natural soil have been studied in the body of the earthworm by means of soil cultivation tests 7, 14, 21 and 28 days after exposure. The results indicated that, in general, superoxide dismutase (SOD) activity, malondialdehyde (MDA) content, metallothionein (MT) content, the expression of heat shock protein 70 (HSP 70) and all the tested geno-toxicity parameters are promoted as time elapses and with increasing concentration of DEHP. However, peroxidase (POD) activity, neutral red retention time (NRRT) and mitochondrial membrane potential difference values were found to decrease even at a low concentration of DEHP of 1 mg kg^-1^ soil (*p*<0.05). Clear toxic effects of DEHP on *E*. *fetida* have been generally recognized by means of the disturbance of antioxidant enzyme activity/content and critical proteins, cell membrane and organelle disorder and DNA damage estimated by length of tail, tail DNA ratio, and tail moment parameters. A concentration of DEHP of 3 mg kg^-1^ may be recommended as a precaution against the potential risk of PAEs in soils and for indicating suitable threshold values for other soil animals and soil micro-organisms.

## Introduction

Phthalic acid esters (PAEs) are a family of ubiquitous chemical additives used as plasticizers, mainly in polyvinylchloride (PVC) products [1]. They are combined physically rather than chemically to the polymer structure so that PAE compounds can be readily released into the environment in relatively large quantities during the extensive use of PVC products [2]. Di-(2-ethylhexyl) phthalate (DEHP), one of the most frequently detected PAE compounds in contaminated environments, can induce testicular damage, decrease fertility, and decrease sperm motility in both developing and adult rodents as a reproductive and developmental toxicant and endocrine disruptor, and can increase the incidence of a range of reproductive defects including cryptorchidism and hypospadias in newborn boys and act as a promoter of testicular cancer, thus representing an important risk to the environment and to human health [3, 4]. Due its suspect carcinogenicity, estrogenicity and influence on the human immune system [3, 5], DEHP was nominated a prior control pollutant and environmental hormone together with five other representative PAE compounds by the US Environmental Protection Agency (USEPA) in 1999. In May 2011 the illegal use of the phthalate plasticizer DEHP in clouding agents used as additives in foods and beverages reported in Taiwan drew attention to DEHP as a problem and a major public health issue [6]. DEHP accounts for about 50% of the total production of PAEs and is also the most widely studied high-molecular-weight PAE compound. High concentrations of DEHP have been detected in different environmental matrices including soils and sediments, the final sinks, in which concentrations range from 0.2 to about 25.2 mg kg^-1^ [7]. High lipophicity and resistance to degradation (> 2000 years under aqueous hydrolysis) have contributed to its residual and cumulative environmental problems [8].

Earthworms are typical saprozoic soil invertebrates and are key organisms for soil functioning. They can be exposed to pollutants entering the soil through both dermal contact and ingestion [9]. Currently, in both types of coelomocyte populations, amoebocytes are more responsive and persuasive for all mitotic figures observed in terms of oxidative stress, cyto-toxicity and geno-toxicity tests of soil pollutants [10]. In addition to the standard eco-toxicity tests employing mortality and growth, more detectable responses in antioxidant chemicals, namely peroxidase (POD) activity, superoxide dismutase (SOD) activity, and malondialdehyde (MDA) content may be used as reliable biochemical markers in the toxicity evaluation of pollutants to assess their importance in anti-oxidative defense [11, 12]. Flow cytometry (FCM) enables the rapid analysis of structural characteristics of cells without staining or rapid determination when cells are stained and compared to traditional assays, and FCM provides the statistical results of > 10 000 individual cells rather than a mean value tested at a population level, excluding the influence of individual differences efficiently in eco-toxicity assays [13, 14]. In investigating geno-toxicity effects to earthworms, the comet assay is always sensitive and reliable, especially when parameters such as length of tail, tail DNA ratio, and tail moment are employed [15]. Earthworms have been widely used in the investigation of adverse effects of persistent organic pollutants (POPs), and recently of effects of PAEs in natural soil to *Eisenia fetida* [10, 15]. Enhanced frequency of apoptosis has been employed in previous studies on organisms including earthworms with satisfactory results [9, 16]. Other popular biomarkers including neutral red retention time (NRRT), metallothionein (MT) and heat shock proteins (HSP 70) are usually chosen to describe the mechanisms by which pollutants damage target earthworms in tests using spiked soil [17, 18]. The FCM assay, the intuition and accuracy of the comet assay and the specificity of NRRT, MT and HSP determination were all used in the current investigation.

In the present study we investigated some effects of DEHP on *E*. *fetida* in natural soil spiked with different concentrations of DEHP during a cultivation period of 28 days. Variation in antioxidant enzymes monitored by total protein content, SOD activity, POD activity and MDA content, and cyto-toxicity including changes in NRRT, intracellular Ca^2+^ concentrations, potential difference value of the mitochondrial membrane, MT content, the expression of HSP 70 and geno-toxicity determined by the comet assay were all compared to reveal microcosm damage effects. The DEHP concentration in contaminated soil deserves special attention and reliable indicators were used to understand the toxicity mechanisms of DEHP to the model earthworm *E*. *fetida* in the present study.

## Methods and materials

### Chemicals and reagents

Di-(2-ethylhexyl) phthalate (DEHP, 99.6%) was obtained from AccuStandard, Inc., New Haven, CT. Assay kits (Catalog numbers A001-1 for SOD, A084-1 for POD, A045-3 for BCA/ total protein content and A003-1 for MDA) were purchased from Jiancheng Bioengineering Institute, Nanjing, East China. The MT Elisa kit 48T for earthworms was purchased from RD Systems China Co. Ltd., Shanghai, China. The Dako LSAB peroxidase kit was obtained from Dako A/S, Glostrup, Denmark. The Fluo-3/AM kit for intracellular Ca^2+^ concentration and the Rhodamine 123 kit for mitochondrial membrane potential difference were sourced from Becton Dickinson/BD Biosciences Pharmingen, San Diego, CA. Guaiacol glyceryl ether (≥98%, C_10_H_14_O_4_), monoclonal mouse anti-human anti-HSP 70, Triton X and bovine serum albumin (BSA) were obtained from Sigma Chemical Co., St. Louis, MO. All other chemicals used were analytical grade reagents purchased from the National Pharmaceutical Group Chemical Reagent Co., Ltd., Shanghai, China.

### Quantitative analysis of DEHP

The analysis of DEHP concentrations in soil after incubation for 28 days was carried out according to Ma et al [19]. DEHP in 10 g of soil was extracted with a total volume of 70 mL acetone:hexane (1:1 v/v) three times in a water bath at 25°C and reduced in the flask by rotary evaporation to 1–2 mL (350 mbar, 40°C water bath, 80 rpm) after centrifugation at 1 500 rpm. Column chromatography purification was performed in a glass column (1 × 26 cm) with Na_2_SO_4_, neutral Al_2_O_3_ and neutral silica gel (from bottom to top) with acetone:hexane (1:4 v/v) before collection and reduction to < 1 mL by rotary evaporation as described above.

Analysis was performed with an Agilent 7890GC-5975 MSD gas chromatograph-mass spectrometer. During analysis, whole procedure blanks, soil matrix blanks, spiked soil matrix and parallel samples were all employed together with analysis of the certified reference material to ensure quality control.

### Earthworms and soil preparation

*Eisenia fetida* individuals purchased from a farm in Nanjing, East China were selected as the test organism. They were held for one month in the laboratory and were fed with cattle feces at 20 ± 1°C at a moisture content of about 50%. Healthy strong adult earthworms with a visible clitellum (350 ± 50 mg) were selected 24 h prior to use and their gut was purged.

A typical yellow brown soil classified as Alfisols according to the USDA soil classification with a pH (in water) of 7.4 was collected from the top 15 cm of a seldom disturbed mountain area (32° 8' 51" N, 118° 57' 58" E) at Qixia District, Nanjing, Jiangsu Province, East China. The soil has a clay content of 1.67 g kg^-1^, an organic matter content of 14.6 g kg^-1^, and available nitrogen, phosphorus and potassium concentrations of 96.8, 14.4 and 102.8 mg kg^-1^, respectively. The soil was passed through a 2 mm sieve prior to the incubation test and the soil background concentration of DEHP was determined to be 0.087 ± 0.004 mg kg^-1^.

Five-mL aliquots of DEHP stock solutions at 200, 600, 1800 and 5400 mg L^-1^ were diluted to 10 mL with acetone for the toxicity test and sprayed on the surface of 1-kg aliquots of prepared soil and thoroughly mixed until the acetone evaporated to obtain final concentrations of about 1, 3, 9 and 27 mg DEHP kg^-1^ soil. Each spiked soil was placed in a brown reagent jar and kept at about 25 ± 1°C. Controls (0 mg kg^-1^) were also prepared using 10 mL of acetone without DEHP to avoid errors due to the solvent. Four replicate jars of the control and each DEHP concentration treatment were set up containing 1 kg of soil and 20 earthworms, giving a total of 400 test worms. On days 7, 14, 21, and 28 during the incubation period four worms were collected from each replicate jar for measurement and analysis.

### Harvesting of enzyme solution and coelomocytes

Randomly selected earthworms in each jar were subjected to enzyme extraction and harvesting of coelomocytes as described by Ma et al. [10].

### Biochemical assays

#### Antioxidant determination

Total protein content, SOD and POD activities and MDA content were determined according to the instructions in the kits used (A045-3, A001-1, A084-1 and A003-1). The enzyme extract solution collected for SODase assay was assayed for its ability to inhibit the photochemical reduction of NBT. One unit of SODase activity is defined as the amount of enzyme inhibiting 50% of the initial reduction of NBT under light. Enzymatic activity is expressed in unit (U) per milligram FW.

The POD assay was carried out based on the observation that POD can catalyze the transformation of guaiacol to tetraguaiacol (a brown colored product) in the presence of H_2_O_2_. Changes in absorbance of the reaction solution at 470 nm were determined every 60 s. POD activity is expressed by change in absorbance as U g^-1^ FW min^-1^.

Two mL of collected enzyme solution were added to 2 mL of 0.5% TBA in 20% TCA and heated in a boiling water bath for 30 min. The mixture was centrifuged at 3 996 ×*g* for 5 min and immediately cooled on ice. The absorbance of the supernatant at 450, 532 and 600 nm was recorded and the MDA content was calculated.

#### Neutral-red retention time (NRRT) assay

The measurement and counting of the neutral-red retention time followed the method of Ma et al. [10] using FCM. A coelomic fluid sample was mixed with a balanced salt solution containing a working NR solution of 80 μg mL^-1^ (1:1) and determined on FCM every minute for two hours. The NRRT is the time of the first interval where the ratio of coelomocytes with fully stained cytoplasm exceeds 50% of the total number of coelomocytes counted.

#### Metallothionein (MT)

The determination of MT contents followed the instructions in the earthworm MT-Elisa kit. The animals were placed in 3 mL ice-cold Tris-HCl 25 mmol buffer (pH 7.2 at 20°C), 0.1 mmol phenylmethylsulfonyl fluoride as antiprotease and 0.5 mmol dithiothreitol as the sulfydryl-protecting agent to prevent oxidation of the MT and then homogenized with distilled water. The homogenate was centrifuged at 10 000 ×*g* for 20 min at 4°C in individual 1.5 mL microtubes. The purified earthworm MT antibody was used to coat the micro pores before combination with MT antibody was labeled with horseradish peroxidase. The combined compounds were thorough washed and colored with tetramethyl benzidine as substrate. The optical density value of the mixture at 450 nm was recorded and used to calculate the concentration of MT.

#### Heat shock proteins (HSP 70)

HSP 70 in extruded coelomocytes of treated *E*. *fetida* were sampled and determined by western blot on day 28 after exposure to spiked natural soil. The immune-peroxidase staining protocol was used on methanol-fixed cytospin preparations after blocking of endogenous peroxidase with 3% H_2_O_2_ as described previously [20]. Monoclonal mouse anti-human anti-HSP 70 were diluted 1:1000 with 0.25% Triton X in phosphate buffer supplemented with 1% BSA (overnight, 4°C) followed by the labeled streptavidin biotin method (Dako LSAB peroxidase kit).

#### Intracellular Ca^2+^ concentrations and mitochondrial membrane potential difference

Concentrations of intracellular calcium ions and potential difference in the mitochondrial membrane (ΔΨm) were measured by FL2 fluorescence intensity determined by flow cytometry assays with sampling and determination on day 28 after exposure in spiked natural soil.

The harvested coelomocytes were collected and washed once in PBS. The cells were then re-suspended in 0.5 mL PBS. Fluo-3/AM solution (final concentration 5 μM) was added for evaluation of Ca^2+^ concentration. A Rhodamine 123 solution (final concentration 10 μg mL^-1^) was added for mitochondrial membrane potential evaluation. The cells were incubated in darkness at 37°C for 40 min. After incubation the cells were washed once and re-suspended in PBS. The fluorescence intensity was analyzed by flow cytometry and > 10000 events were captured for every analysis [21].

#### Comet assay

The comet assay was performed according to Ma et al. [10]. The harvested coelomocyte cell suspension was mixed with 100 mL of 0.7% low melting agar (LMA) in PBS at 37°C and pipetted onto fully frosted slides pre-coated with a layer of 100 mL 0.8% normal melting agar (NMA). After solidification on ice, a further layer of 85 mL LMA was added and the slides were immersed in a lysis solution (2.5 M NaCl, 10 mM Tris, 100 mM Na_2_-EDTA (pH 10.0), 1% Na-sarcosinate, 10% dimethyl sulfoxide (DMSO), and 1% Triton X-100). The slides were then incubated in an electrophoresis tank containing 300 mM NaOH with 1 mM Na_2_-EDTA for 20 min prior to electrophoresis for 15 min at 25 V (300 mA). The slides were then neutralized (0.4 M Tris, pH 7.5) three times at 5-min intervals and stained with 40 mL ethidium bromide (13 mg mL^-1^) for fluorescence microscopy analysis which was performed with a Zeiss Axiovert 40 (Carl Zeiss AG, Oberkochen, Germany) using a charge coupled device (CCD) digital imaging system. The images of the comet assay were analyzed last using CASP. One hundred cell nuclei on each slide were analyzed. Length of tail, tail DNA ratio, tail moment and olive tail moment were used for the quantification of DNA damage. From repeated experiments the average median tail moment value was calculated for each treatment group from the median tail moment value of each slide.

### Statistical analysis

All values are presented as mean ± standard error of the mean (SEM). One-way analysis of variance and the least significant difference test were performed using the SPSS software (SPSS 13.0) package and *p< 0.05 and **p<0.01 were considered to be significant and highly significant probability levels, respectively.

## Results and discussion

### Termination concentration of DEHP in soil

We selected the range of DEHP concentrations used in this experiment based on the average concentrations detected in Chinese soils which are reported to range between 0.2 and 25.2 mg kg^-1^ [22]. After incubation for 28 days the soils spiked with 0, 1, 3, 9 and 27 mg DEHP kg^-1^ soil contained 0.026 ± 0.005, 0.709 ± 0.005, 2.573 ± 0.009, 7.887 ± 0.008 and 18.929 ± 0.013 mg DEHP kg^-1^ soil, respectively. The DEHP degradation rates were < 30% and therefore DEHP toxicity can be considered to have continued throughout the incubation period. Together with DnBP which was also degraded by > 54% over the same cultivation period and under the same conditions the residual levels of DEHP in soil with earthworms supports the conclusion that the degradation rate decreases with increasing molecular weight of PAEs, although the presence of earthworms may promote the degradation of organic matter [10, 23, 24].

### Critical parameters in spiked soil tests

#### Total protein content

**Fig 1:**
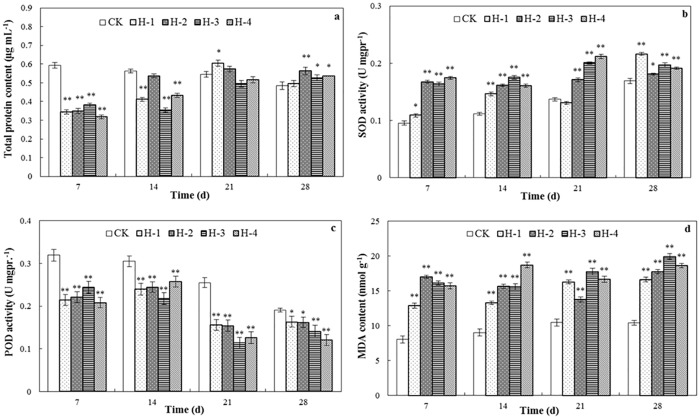
Toxicity effects of DEHP on biochemical properties of *E*. *fetida* in the spiked soil test. (a) total protein content; (b) SOD activity; (c) POD activity; and (d) MDA content were determined after treatment for 7, 14, 21 and 28 d in spiked natural soil CK, H-1, H-2, H-3, and H-4 (n = 4; error bars, SEM/mean value of standard errors). The spiked concentrations of DEHP were 0, 1, 3, 9 and 27 mg kg^-1^ soil. Asterisks show significant differences at *p*<0.05 level compared to the control; double asterisks show significant differences at *p*<0.01 level compared to the control (S1 Data).

Total protein content reflects the sum of all types of enzymatic and non-enzymatic cellular proteins. Fig 1A shows that the total protein contents in the different spiked treatments were significantly lower than in the control on day 7 (*p*<0.01), possibly due to both the consumption of different enzymes in cellular protection under contamination stress and the inhibition of protein synthesis when earthworms were exposed to DEHP at different concentrations [10]. However, after the physical adaptation of the earthworm bodies the total protein contents in the different treatments increased above that of the control, especially when the DEHP concentration was > 3 mg DEHP kg^-1^ soil (*p*<0.05).

#### SOD and POD activities

SOD in earthworms is sometimes induced and a dynamic balance is maintained to meet the needs of organisms to eliminate O^2-^ but the balance can be easily disrupted by the stress of pollutants. Fig 1B shows increasing SOD activity with increasing time and increasing of concentration of spiked DEHP (*p*<0.05), with a maximum value on day 28 in the 1 mg kg^-1^ treatment. However, in studies of human testicular damage DEHP has decreased or inhibited the activity level of SOD [25, 26]. Published results of other studies indicate that endocrine disrupting compounds can disturb the anti-oxidative balance in wildlife vertebrates [27], with similar results in invertebrates in the current experiment. Irrespective of the mechanism in earthworms, the control of DEHP may lead to lower oxidative stress.

The variation in POD activity was showed the opposite to that of SOD activity which was significantly lower than in the control (*p*<0.05) at different concentrations of spiked DEHP and cultivation times (Fig 1C). POD also catalyzes the oxidation of numerous substrates in the presence of H_2_O_2_ to form water molecules and a decrease in POD activity indicates the consumption of harmful H_2_O_2_ in cells.

#### MDA content

MDA is the product of lipid peroxidation and has been used to assess oxidative stress in earthworms [28]. An increase in MDA content usually indicates protective mechanisms and the consumption of MDA indicates slight damage to organs and both trends can appear simultaneously in different parts of the same organism treated with DEHP [29]. As shown in Fig 1D, MDA content was stimulated (*p*<0.01) at different DEHP addition levels compared with the control but without a significant linear relationship with increasing DEHP concentration. MDA is considered to be a sensitive parameter which may indirectly reflect the degree of intracellular oxidative injury as it is the product of reaction between unsaturated fatty acids and free radicals in cellular membranes, and one of the most damaging effects of reactive oxygen species and their products in cells is the peroxidation of membrane lipids which can be indicated by the detection of MDA [30, 31]. Higher MDA content than the control value in the present study indicates oxidative damage in earthworms as reported previously in the testicular tissues of rats [25].

#### NRRT assay

**Fig 2:**
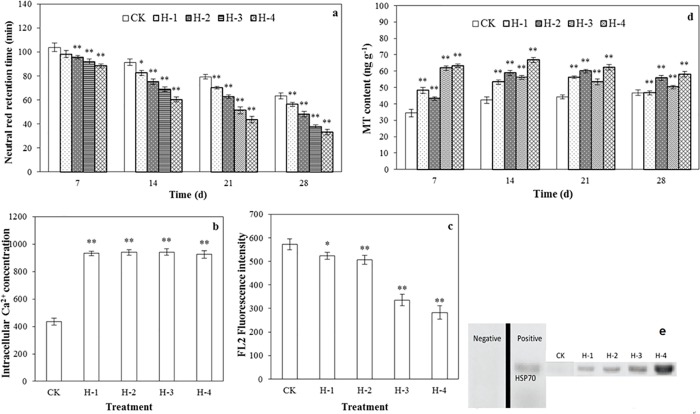
Cyto-toxicity of DEHP to *E*. *fetida* in the spiked soil test. (a) lysosomal membrane stability characterized by neutral red retention time (NRRT), (b) concentration of intracellular calcium ions and (c) potential difference in the mitochondrial membrane (ΔΨm) measured by FL2 fluorescence intensity determined by flow cytometry assays; (d) metallothionein (MT) level determined with assay kits; and (e) heat shock proteins (HSP 70) determined by western blot in extruded coelomocytes of treated *E*. *fetida*. (a) and (d) were determined on days 7, 14, 21 and 28; (b), (c) and (e) were determined on day 28 day after exposure in spiked natural soil; CK, H-1, H-2, H-3, and H-4 (n = 4; error bars, SEM). Refer to Fig 1 for details of other annotations (S1 Data).

Lysosomal membrane stability is a general subcellular biomarker for the action of toxic pollutants that has been found to be reliable and dose-related in different earthworm species, and the employment of flow cytometry measurement of the neutral red retention time in earthworm coelomocytes has increased in popularity [10]. Alternations of the lasosomal system provide an early response to contaminant exposure in earthworms and former studies have demonstrated the usefulness of this parameter as a predictor of adverse effects [32]. In the present study the values of NRRT of the DEHP treatments were significantly lower than the control at different spiked concentrations and time points, especially when the concentration of DEHP exceeded 3 mg kg^-1^ (*p*<0.01) (Fig 2A). The dysfunction of the lysosomal membrane may be recognized as a potential mechanism of DEHP toxicity in earthworms [33].

#### Intracellular Ca^2+^ concentration and mitochondrial membrane potential difference (ΔΨm)

The intracellular Ca^2+^ concentration in the coelomocytes was tested using Fluo-3/AM to investigate the effect of DEHP on Ca^2+^ influx, and there were highly significant increases (*p*<0.01) in the different treatments compared to the control at the end of the incubation period (Fig 2B). In general, calcium in many cell types is a universal second messenger of signal transduction, and the application of flow cytometry-based assays to measure intracellular calcium levels in the coelomocytes of *E*. *fetida* has been increasingly used in recent investigations [34]. It has been found that DEHP increases the intracellular calcium concentration by inducing a Ca^2+^ influx from the extracellular medium in human granulocytes [35], and a significant increase in intracellular Ca^2+^ concentration might be associated with the NRRT results because the loss of lysosomal membrane stability may make facilitate the entry of the coelomocytes by calcium ions and lead to further disruption of signal transduction.

According to Fig 2C significant differences in mitochondrial membrane potential difference (ΔΨm) were revealed by FL2 fluorescence intensity (*p*<0.05) in the different DEHP spiked treatments compared to the control. DEHP administration significantly lowered the testicular mitochondrial viability in rats [25]. A decrease in mitochondrial membrane potential difference demonstrates the instability of the coelomocytes after treatment with different concentrations of DEHP, and the mitochondrial membrane damage induced by DEHP may be a further explanation for membrane polarization and cell death [36]. On the level of cyto-toxicity both intracellular Ca^2+^ concentration and mitochondrial membrane potential difference may be selected as biological indicators in future estimation of DEHP eco-toxicity in earthworms.

#### MT content

MTs are low-molecular-weight cysteine-rich metal-binding proteins that participate in an array of cellular protective stress responses and play an important role in the cell as scavengers of free radicals [37]. The MT contents were significantly higher (*p*<0.01) in earthworms from all the DEHP addition treatments than in the control with maximum values in the 27 mg kg^-1^ treatment at different incubation times (Fig 2D). Higher levels of target pollutants in soil have resulted in the clear promotion of MT content although positive correlations between MT content and DEHP concentration in all earthworms have not always been observed. MTs have been widely accepted as efficient biomarkers for monitoring soil contamination, especially for metals and metalloids and they play a vital role in the detoxification pathway of pollutants [38]. However, in the present study substantial promotion of MT content has been detected.

#### Expression of HSP 70

Protein HSP from earthworms or other organisms induced under pollution stress, and especially HSP 70, have been explored as a biomarker of the soil ecosystem. HSP may be expressed in response to different physical or chemical conditions, rather than simply with or without heat shock, but DEHP may stimulate the over expression of HSP with higher molecular weights [39]. The increase in total protein content on day 28 (Fig 1A) is in general accordance with results of some other representative proteins such as the expression of the amount of HSP 70, and a possible mechanistic relationship may be investigated in future studies. In the present study the amount of HSP 70 expressed was directly stimulated with increasing test DEHP concentrations in spiked soils (Fig 2E), suggesting the possibility of selecting HSP 70 as the target sensitive bio-indicator for DEHP contaminated soils. Constitutive expression of HSP 70 was found in coelomocytes from control worms and the early detectability of altered levels of specific gene expression products (MT and HSPs) highlights the sensitivity and predictive possibilities of these (and similar) molecular biomarkers [18].

#### Comet assay

The comet assay is widely used in ecotoxicology to detect the level of primary DNA damage and the damage to DNA in a single coelomocyte may be assessed directly [40]. In the investigation of the oxidative stress potential of DEHP and its consequences in testicular cells, geno-toxicity potential has been found to be clearly affected although its main metabolite, mono-(2-ethylhexyl)-phthalate (MEHP), was found to be more potent than the parent compound [41].

**Fig 3:**
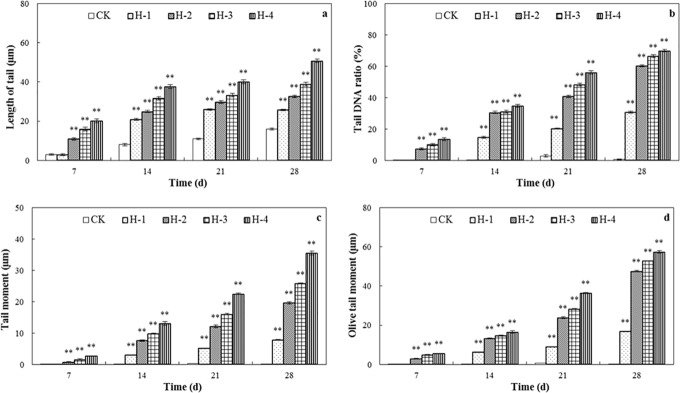
Geno-toxicity of DEHP determined by comet assay in the spiked soil test. (a) length of tail; (b) tail DNA ratio; (c) tail moment and (d) olive tail moment of coelomocytes in treated *E*. *fetida* after treatment for 7, 14, 21 and 28 d in spiked natural soil; CK, H-1, H-2, H-3, and H-4 (n = 4; error bars, SEM). Length of tail (TL) denotes tail length in arbitrary units; tail DNA ratio shows relative ratio of DNA in the comet tail; tail moment (TM) represents the integrated value of DNA density multiplied by the migration distance; and Olive tail moment (OTM) is the product of the distance between the center of gravity of the head and the center of gravity of the tail and percent tail DNA. Refer to Fig 1 for other annotations (S1 Data).

In Fig 3, significant DNA damage was observed in earthworms during our 28-day experiment in all four DEHP spiked treatments after day 14 (*p*<0.01) with clear correlations between DNA damage and both DEHP concentrations and exposure time. The length of tail values increased over three times compared with the control in the 27 mg DEHP kg^-1^ soil treatment at different sampling times, and the tail DNA ratio by up to 70% by the end of the incubation period. DEHP damage to DNA in other organisms has been reported, including significant induction of the Olive tail moment (OTM) of mouse hepatocytes compared with the negative control with a dose-response relationship [42].

## Conclusions

Distinctive toxicity effects on *E*. *fetida* under different DEHP stress levels in soil were generally indicated by levels of enzyme activity/content impact, intracellular disorder and DNA damage, in terms of increasing of total protein content, SOD activity, MDA content, MT content, intracellular Ca^2+^ concentration, HSP 70 expression and all the comet assay results and the lowering of POD activity, NRRT and mitochondrial membrane potential difference at different spiked DEHP concentrations (*p*<0.05). In the present study the results suggest the possibility of using MT contents in the investigation of toxicity to earthworms of organic pollutants such as DEHP rather than that of the common metal and metalloid pollutants in contaminated soils (S1 Highlights). The toxic effects of DEHP in soil to earthworms might be derived from damage to membranes of intracellular organelles, then to the intracellular macromolecular DNA, and finally to protein molecules including different protein indicators and antioxidant enzymes. As a precautionary level for potential risk of PAE chemicals in soils, 3 mg kg^-1^ DEHP may be recommended as a potentially useful indicative concentration for highly significant changes in the different parameters tested.

## Supporting information

S1 Data Supporting data of all figures. (XLSX)

S1 Highlights Highlights. (DOC)
