# Supplementary material for: Toxicity effects of di-(2-ethylhexyl) phthalate to Eisenia fetida at enzyme, cellular and genetic levels
Source: PLoS One. 2017 Mar 20;12(3):e0173957. doi: 10.1371/journal.pone.0173957 (PMC5358789; doi:10.1371/journal.pone.0173957)
Supplement: S1 Highlights — (DOC) [file pone.0173957.s002.doc]

Highlights

DEHP toxicity to *Eisenia fetida* in spiked soil was investigated.

Metallothionein could be used as a sensitive bio-indicator.

Membrane damage might be the critical step for coelomocyte apoptosis.

DNA in coelomocytes could also be damaged by DEHP.

3 mg kg^-1^ DEHP could be recommended as a threshold concentration.
